# Tribological properties of MAO ceramic coatings with annulus array texture on disposable surgical gloves

**DOI:** 10.3389/fbioe.2024.1397050

**Published:** 2024-05-01

**Authors:** Jing Ji, Zhenbo Bai, Jinfeng Wang, Huiyun Yang, Hailin Lu, Jing Fang

**Affiliations:** ^1^ Department of Gynecology and Obstetrics, The First Affiliated Hospital of Xi’an Jiaotong University, Xi’an, Shaanxi, China; ^2^ Group of Mechanical and Biomedical Engineering, College of Mechanical and Electronic Engineering, Xi’an Polytechnic University, Xi’an, Shaanxi, China

**Keywords:** annulus array texture, micro-arc oxidation, coefficient of friction, disposable surgical gloves, biomaterials

## Abstract

**Introduction:** In recent research, the expansion in the use of Mg alloys for biomedical applications has been approached by modifying their surfaces in conjunction with micro-arc oxidation (MAO) techniques which enhance their abrasion and corrosion resistance.

**Methods:** In this study, combining laser texturing and MAO techniques to produce the dense ceramic coatings with microstructures. On the surface of the AZ31 Mg alloy, a micro-raised annulus array texture has been designed in order to increase the surface friction under liquid lubrication and to improve the operator’s grip when holding the tool. For this work, the micro-morphology of the coatings was characterised, and the friction properties of the commonly used scalpel shank material 316 L, the untextured surface and the textured surface were comparatively analysed against disposable surgical gloves.

**Results and discussion:** The results show that the Laser-MAO ceramic coating grows homogenous, the porosity decreases from 14.3% to 7.8%, and the morphology after friction indicates that the coating has good wear resistance. More specifically, the average coefficient of friction (COF) of the three types of gloves coated with Laser-MAO ceramic was higher than that of the 316 L and MAO ceramic coatings under the action of the annulus-integrated texture under the lubrication conditions of physiological saline and defatted sheep blood, which achieved the goal of increasing friction for the purpose of helping to prevent the problem of tool slippage from the hand.

## 1 Introduction

With the development of modern medical technology, the scalpel has been transformed from the original single-piece instrument to a separate blade and handle tool (Barbosa, 2022). It helps in conveniently changing the blades and keeping the blades sharp. Currently, the scalpel handle acts as a pivot to support and load the blade, and its research direction focuses, on the one hand, on the improvement of the shape of the handle to be suitable for different departments and the needs of the surgery (Mohan et al., 2022). On the other hand, it focuses on the improvement of the connection between the blade and handle to meet the new requirement of quick and easy blade change while ensuring stability (Su et al., 2023). The meticulous categorization of knife handles has been a great help to doctors in performing surgical procedures, but it seems that less research has been performed on the materials used for knife handles. The most used handles are divided into disposable plastic handles and reusable stainless steel handles (Zaidi et al., 2021). The former meets the convenience and safety requirements, while the latter requires regular maintenance and sterilization and is rust and corrosion-resistant. As a high-performance lightweight structural material, Mg alloys have a specific gravity similar to that of plastics and are as stiff and strong as aluminum (Al). At the same time, they have good casting and dimensional stability (Cai et al., 2019; Wang et al., 2021). In the literature, it has been shown that Mg ions, as an essential trace element, have the ability to promote osteoblast differentiation and inhibit bacterial activity in an ionic form (Xue et al., 2023). Owing to their excellent biocompatibility and mechanical properties, Mg alloys are the material of choice for bone implants and play a crucial role in the biomedical industry (Butt et al., 2020; Li et al., 2020). Therefore, it is of cutting-edge importance to introduce a lightweight and high-performance material for use in scalpel handles.

Although Mg alloy materials have the advantage of possessing unique properties, in physiological environments such as body fluids, the Mg alloy will be corroded, and as a result, the mechanical properties will be reduced. Therefore, surface treatment of Mg alloy materials is very necessary (Li et al., 2018; Kaseem et al., 2023). Stojanović et al. (2023) processed Mg alloy materials using a reference composite casting process to form composites with better wear resistance. Micro-arc oxidation (MAO) technology has been developed from anodic oxidation technology and is superior to anodic oxidation for the preparation of ceramic coatings on valve metals (Xue et al., 2019). During the MAO process, a micro-arc discharge is generated on the surface of the Mg alloy at high temperatures and pressures, which results in the combination of Mg atoms in the substrate micro-regions with an oxygen plasma, producing a ceramic oxide coating (Hui et al., 2020). The research shows that MAO ceramic coating greatly improves the surface hardness, wear resistance, heat resistance, and corrosion resistance of the material, which fundamentally overcomes the shortcomings of AL, Mg, and Ti-alloy materials in the application. At the same time, the process is stable and reliable, has simple and easy-to-operate equipment, and is a green, environmentally friendly material surface treatment technology (de Castro et al., 2018; Yan et al., 2024). Many studies have shown that the microstructure and properties of MAO coatings on Mg alloys mainly depend on the electrolyte, electrical parameters, and substrate composition during processing (Rúa et al., 2019; Yuan et al., 2021). It has been confirmed that anions in the electrolyte and other alloying atoms in the Mg substrate are involved in the micro-arc discharge process, which affects the properties of the coating such as breakdown voltage, microstructure, composition, and growth rate (Kaseem et al., 2021). Jian et al. (2019) prepared coatings with favorable corrosion resistance in electrolytes containing silicon and phosphorus, while in vivo experiments were conducted on magnesium alloy-based screws, and the MAO layer was well adhered, which effectively reduced the degradation rate of the screws and facilitated bone healing. The MAO technology has excellent surface modification capabilities, improving the biocompatibility and bioactivity, thermal stability, and dielectric properties of the materials and enhancing the potential applications of magnesium alloys in biomedicine (Aktug et al., 2019).

In the development of laser technology, laser texturing on surfaces has been used to investigate how to reduce the coefficient of friction (COF) on surfaces (Zhan et al., 2019; Wan et al., 2021). However, rough surfaces have the natural advantage of increasing the interaction force between the friction surfaces, which serves to increase friction (Babic et al., 2019; Jain and Bajpai, 2022). Developments in the field of bionics promote advances in laser surface texturing (LST) technology (Vencl et al., 2019; Amziane et al., 2023). Ivanović et al. (2018) investigated tribological properties in the field of biomimicry at the micro and nano levels, which showed the great potential of technological innovations and the value of engineering applications. Meanwhile, Wang et al. (2019a) and Nashrah et al. (2019) showed that in silicate-based electrolytes, the grooved samples reached the breakdown voltage earlier than the flat samples, showing slightly faster growth rates and thicker coatings. Li et al. (2019) proposed a one-stop laser surface texturing method, which reduced the density of coating micropores and increased the bond strength of the coating by 35.7%. Laser texturing technology has a series of advantages, such as high processing precision, high efficiency, and a wide range of applications. Moreover, micro-arc oxidation technology is capable of processing surfaces with complex structures, with little requirement for the shape of the substrate. Thus, it can be seen that the combining process proposed in this study is highly implementable and simple to operate.

In this study, Mg alloy was used as a raw material and pretreated by laser texturing technique, and then MAO ceramic coating was manufactured on the metal surface on which the microstructure, friction properties, and corrosion resistance of the coating were investigated. The purpose of this study is to develop a micro-arc oxidized ceramic coating process with micro-structures for application on medical scalpel handles to expand the application field of Mg alloy materials and increase the range of material choices for scalpel handles.

## 2 Materials and methods

### 2.1 Materials

This experiment used commercially available AZ31 magnesium alloy as the raw material, and the size of the disk was Φ30 mm × 3 mm. Sodium silicate (Na_2_SiO_3_), sodium chloride (NaCl), and potassium hydroxide (KOH) were purchased from the Tianjin Damao Chemical Reagent Factory (Tianjin, China). Sodium fluoride (NaF) was purchased from the Tianjin Fuchen Chemical Reagent Factory (Tianjin, China). All reagents were of analytical purity (AR). Defatted sheep blood, belonging to non-Newtonian fluids, was purchased from Henan Yuechi Biotechnology Co. (Henan, China), harvested in an aseptic environment, and stored in freshness, which was consistent with the fresh blood appearing in the surgical environment. Three types of commercially disposable sterile rubber surgical gloves were used in the friction experiments, all of which conformed to the GB7543 product standards and technical requirements and passed the product testing to ensure that the products were qualified and harmless. Glove A (branded Gaobang), glove B (branded Medispo), and glove C (branded Intco Canada Inc) were selected. These types of gloves are the most commonly used in medical treatments due to their ease of access in the market and their relatively low cost.

### 2.2 Sample preparation

At the beginning of the experiment, the samples were polished using 2000# sandpaper for 120 s, followed by polishing treatment using a crystalline phase polishing cloth with the addition of a polishing solution for 60 s. The surface roughness (Ra) of the smoothed substrate was 0.09 μm, and testing was performed on the TR-210 Roughness Tester (Beijing Jitai Technology Detection Device Co., Ltd., China). After that, the surface was cleaned for 600 s in the acetone solution by an ultrasonic machine (Laizhou Huaxing Testing Instruments, China) to remove the surface oils and residues. Laser texturing was performed using a laser marking machine (Da Clan Laser Technology Industry Group, China). The parameters of the laser marking machine used were the scaling speed of 1,000 mm/s and the Q frequency of 20 KHz. The rated power was 20 W with a 50% output power. The samples were frozen in a refrigerator for 2 h at −18°C before laser texturing. MAO processing was performed using a bipolar microelectromechanical oxidation power supply (FL7-MAOB60A, Xi’an Juncheng Precision Technology Co., Ltd.). The electrolyte consisted of 15 g/L sodium silicate, 3 g/L potassium hydroxide, and 3 g/L sodium fluoride. The MAO parameters were set to two stages. The first stage processed the samples in a constant current mode with a forward current of 1 A for 600 s. In the second stage, the forward current was reduced to 0.3 A, and the samples were treated for 5 min. The pulse frequency used was 800 HZ, with a 20% duty cycle.

### 2.3 Friction experiment

**FIGURE 1:**
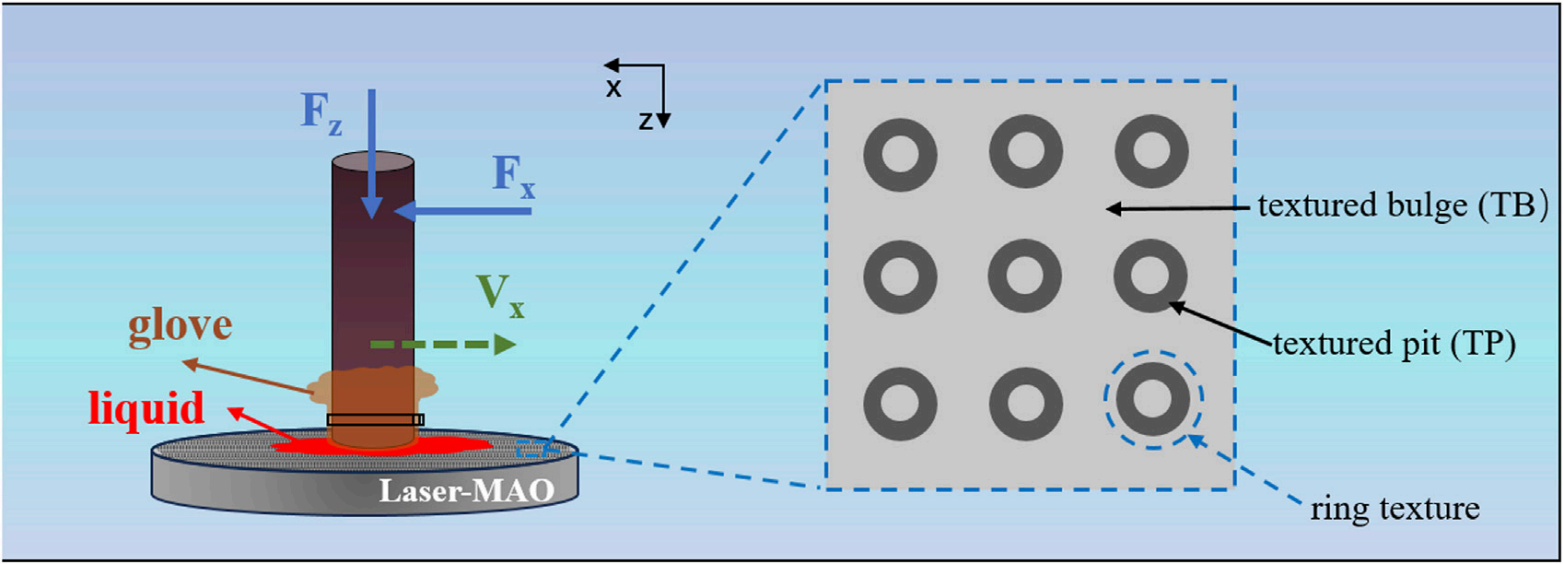
Schematic diagram of the friction experiment.

The friction experiments between the gloves and samples were performed on a GSR-2 Reciprocating Ball and Disk Friction Tester (China Rubber Alcohol Tribometer). With a high-performance sensor (China DY054-A), the instantaneous friction force F was collected and transformed into the electrical input signal to the computer. There was a sensor accuracy of 0.006, and before each test, a friction test using a matched pair with a known coefficient of friction for adjustment of the platform accuracy was carried out. As shown in Figure 1, the sample to be tested was immovable on the working plate of the friction machine. The glove was cut in a square shape (20 mm × 20 mm) and fastened to a round (φ10 mm) loading rod mold using zip ties. A 100 g standard weight was inserted on the loading rod. The motor performed a reciprocating linear movement of the loading bar. The force measured in the opposite direction of sliding was the “friction force F_x_” (the force opposite to the movement), while the applied force was the “normal force F_z_,” which simulated the perpendicular force of a person’s finger pressed downwards on a flat plate. The sliding speed used was 10 mm/s.

In the friction test, three types of disposable sterile rubber surgical gloves, namely, glove A, glove B, and glove C, were used. The parameters of the gloves were provided by the suppliers, and all of them conformed to the industry standards for disposable medical gloves, as shown in Table 1. The test samples were a common surgical knife handle material 316 L, an untextured MAO ceramic-coated sample (MAO), and a laser-textured MAO ceramic-coated sample (Laser-MAO). The test environments were dry friction, physiological saline, and defatted sheep blood to simulate the interaction between surgical gloves and samples under different situations, respectively. The friction experiments were repeated three times for a single type of sample; each friction experiment used a newly prepared sample, and the data were processed by taking the average of the three times to reduce friction inaccuracy.

**TABLE 1:** Material characteristics of different types of gloves.

Sample	Glove A	Glove B	Glove C
Surface morphology	Textured	Textured	Textured
Models	Powder-free	Powder-free	Powder-free
Glove type	Surgical	Surgical	Surgical
Thickness (mm)	0.17–0.26	0.19–0.21	0.29–0.21
Fracture force (N)	17–21.5	17.5–23	17.3–23.3
Percentage elongation after fracture (%)	730–800	760–820	840–876

### 2.4 Surface characterization

The surface morphology of MAO samples and Laser-MAO samples was observed by scanning electron microscopy (SEM) (JSM-6460LV), which demonstrated the growth of the coatings after MAO processing on samples with different structures. The phase compositions of the sample surfaces were obtained by examining their X-ray diffraction (XRD) patterns using a D8 Advanced Diffractometer (Bruker, Germany) with a scanning angle of 5°–90° and a scanning speed of 10°/min. Using an energy spectrometer (EDS, Kevex), the elemental composition and distribution of the coating at the friction trajectory were analyzed after friction in a degreased, defatted sheep blood environment.

## 3 Results and discussion

### 3.1 Analysis by scanning electron microscopy

**FIGURE 2:**
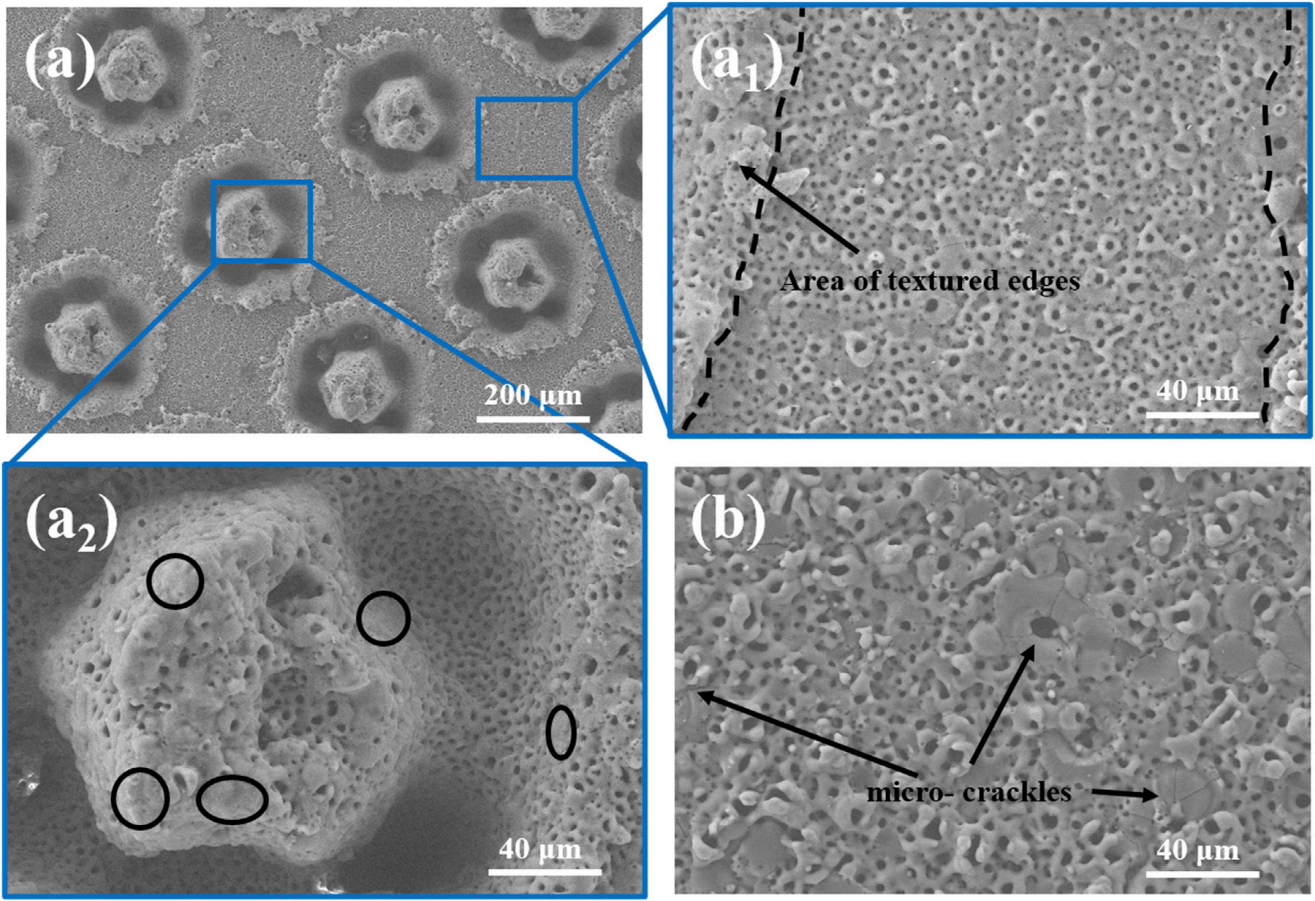
SEM surface morphology for **(A, A1, and A2)** MAO ceramic coating after laser texturing. **(B)** MAO ceramic coating for Mg alloys.

Figure 2A shows the SEM morphology of the ceramic coatings from MAO processing of Mg alloys that had been lasered and textured, which clearly demonstrates the morphology of the texturing and growth of the MAO ceramic coatings. A magnified ×500 surface morphology at a textured bulge (TB) is shown in Figure 2A1. From the ceramic coatings obtained, the typical volcanic pore morphology and a number of characteristic crater-like micropores and microcracks were observed. There is a buildup of the ceramic coating at the edges of the texture pits, which is mainly due to the vaporization of the material upon the formation of the texture pits and liquid-phase explosion that occurs when the material is melted, resulting in the ejection of the magnesium alloy matrix, which condenses in the region of the texture edges (Guan et al., 2014; Zhang et al., 2019). The immersion MAO treatment is able to form ceramic coatings customized to the shape of the workpiece. The workpiece is immersed in the electrolyte, and the whole system is electrically conductive, with the anions in the electrolyte moving toward the metal cations, replenishing the anions consumed by the base to form the coating. Thus, the ceramic coating grows along the Mg alloy stacked at the edges of the texture, preserving the trajectory of the laser-impacted metal material ejected outward. Figure 2A2 shows the surface morphology at the textured pit (TP), magnified at ×500. The ring structure of the texture remains intact, and the laser after-effects have not broken the intermediate columnar structure of the annulus. An interesting aspect of the coating at the TP is the presence of large areas without crater-like features (black circles), which appear as textured crater walls and columnar structures—the areas closest to the high temperature of the laser but not the area where the laser is directly applied. Figure 2B shows the SEM morphology of the ceramic coating obtained by direct MAO treatment of Mg alloy. The sample was not subjected to the high temperature of the laser before MAO treatment, and the micropores were dominated by large diameters with microcracks around to appear. The diameter of crater-like pores was significantly increased in the ceramic coating (TB) after laser texturing treatment and then MAO. Significantly, there is a decrease in large pore-size micropores and the appearance of a large number of small pore-size micropores. Compared to the direct MAO treatment of the substrate, the results at TB indicate that high-temperature treatment of the metal prior to the MAO treatment is useful for the uniform growth of the coating.

**FIGURE 3:**
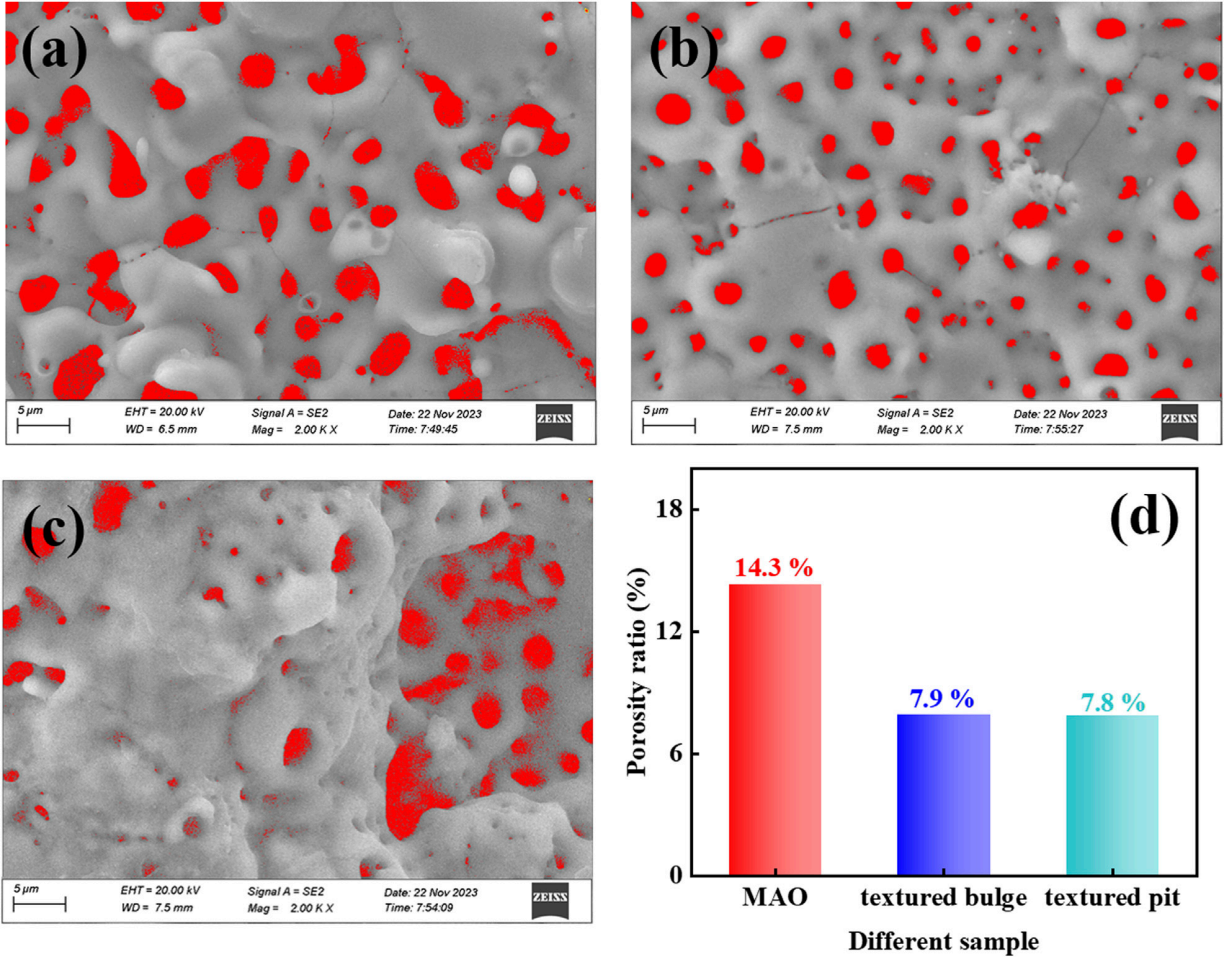
SEM morphology of the coatings at a magnification of ×2000: **(A)** MAO ceramic coating of Mg alloy, and MAO ceramic coating after laser texturing **(B)** at TB and **(C)** at TP; **(D)** surface porosity ratio of the coatings.

Figures 3A–C show the SEM morphology of the MAO ceramic coating of Mg alloy, TB, and TP at a magnification of ×2000, which was colored and processed using image processing software (ImageJ). The micropores and microcracks are well-filled with red. MAO ceramic coating of Mg alloy is characterized by large pore sizes. The ceramic coating at TB after high-temperature influence shows the opposite morphology, where the pores are dominated by small pore sizes and the number of pores increases. At the TP where the laser acts directly, it shows a large diameter in the textured pits, while the textured walls and edges show pores with reduced diameter and number. The porosity of the coatings is shown in Figure 3D, and the coatings that have been laser processed display smaller porosity. A discharge path for the MAO treatment is formed when the micropores are formed, as well as a path for the bubbles generated by the molten oxides and reactions to exclude the surface film. On the one hand, the reduced number of micropores existing in a flat area may be due to the laser being directly applied to the substrate and the Mg element at the edges of the texture having been oxidized to form Mg oxides. The reaction between cations in the substrate and anions is the electrolyte in the first stage of the MAO reaction, which leads to the formation of oxides in the coating. Since oxides of Mg were already present at the TB and at the edge of the TP before MAO processing, the reaction skipped the first stage and entered the second stage. At the interface between the substrate and coating, there is a movement of anions from the electrolyte toward the coating to replenish the anionic vacancies created by the depletion of the substrate, a process that occurs in an oxygen-deficient environment (Wang et al., 2022; Jia et al., 2024). The discharge current of a single micropore was reduced, and the amount of molten oxide discharged instantaneously was decreased (Wu et al., 2023). On the other hand, when MAO processing starts, it will produce instantaneous high temperatures and high pressures in the local area on the surface of the substrate, and the melted MgSiO_3_ and Mg_2_SiO_4_ particles will be mixed with the molten material ejected from the substrate. Under the effect of electrolyte “cold quenching,” the film thickness increases in the local area near the discharge channel, and the discharge channel turns narrower, which results in a dense oxide film with a small microporous size. The area of the TPs may be affected by the grain refinement of the laser-impacted Mg substrate, which would affect the discharge sparking during the MAO processes, manifesting as pores with larger aperture diameters.

### 3.2 Analysis of X-ray diffraction

**FIGURE 4:**
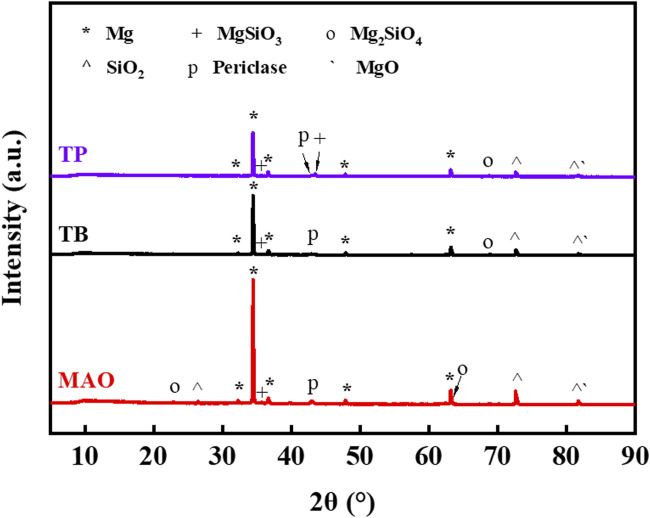
XRD pattern of ceramic coating.

Figure 4 shows the XRD patterns of ceramic coatings prepared from Mg alloys in a silicate electrolyte system. The diffraction peaks of Mg mainly originate from the substrate. Phase identification showed that the coating contained forsterite (Mg_2_SiO_4_), clinoenstatite (MgSiO_3_), periclase (MgO), cristobalite (SiO_2_), and magnesium oxide. Among these, silica originates from silicates in the electrolyte, which are adsorbed by cations in the substrate and react. The equation of reaction (1) (Kaseem et al., 2021) is as follows:

$${\text{SiO}}_{4}^{4‐}\to {\text{SiO}}_{2}.$$ (1)

It further implies that the reaction occurred to produce the rhombohedral system forsterite. Reaction Eq. 2 (Wang et al., 2019b; Liu et al., 2021) is as follows:

$${\text{SiO}}_{2}+2\text{MgO }\to \;{\text{Mg}}_{2}{\text{SiO}}_{4}.$$ (2)

Since the existence of the monoclinic crystal system clinoenstatite, MgO generated in an oxygen-deficient environment has not reacted sufficiently, resulting in an incomplete reaction, reaction Eq. 3 (Yang et al., 2023), as follows:

$${\text{SiO}}_{2}+\text{MgO }\to \;{\text{MgSiO}}_{3}.$$ (3)

The XRD patterns were analyzed, and the peaks at TB and TP were both reduced compared to the MAO coating (Pan et al., 2020). Here, intensity (a.u.) represents the intensity of the diffraction peak, and since the absolute intensity of rays has no practical significance, the relative intensity is used, expressed in arbitrary units. The results show that the ceramic coating formed by laser treatment and MAO is smaller than the original coating.

### 3.3 EDS analysis of laser enameling

**FIGURE 5:**
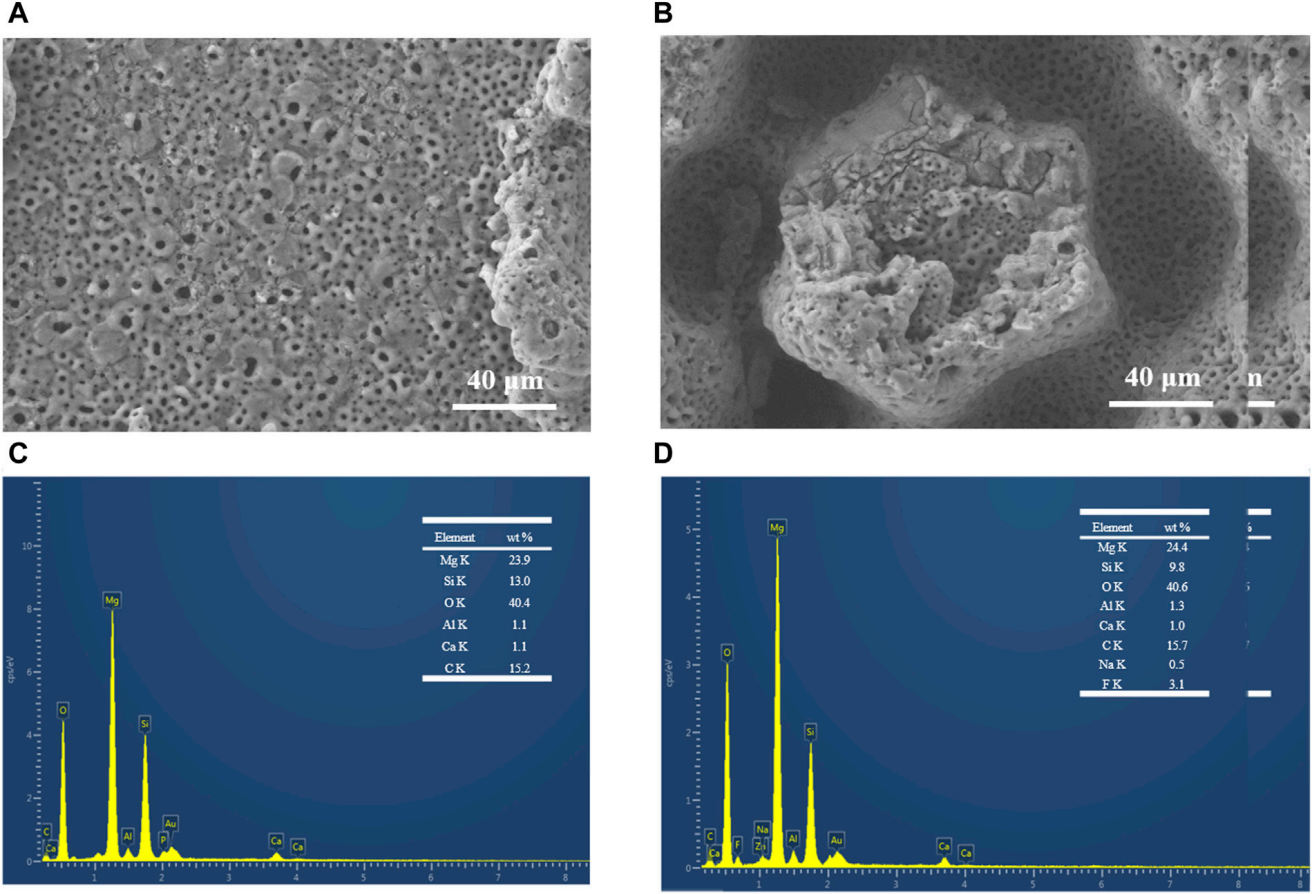
SEM morphology at the friction trajectory of MAO ceramic coatings after the laser texturing process **(A)** at TB and **(B)** at TP; corresponding positional swept EDS elemental analyses **(C)** at TB and **(D)** at TP.

Some of the friction experiments were carried out in a blood environment that simulated the application of the coatings in surgical scenarios to study the performance of the coatings. Figures 5A, B show the SEM morphology of the surface at the friction track after laser texturing processing for MAO ceramic coatings at TB and TP, respectively, under the condition of defatted sheep blood. There is minor wear at the friction track, and yet the coating as a whole is not damaged. Microcracks in the coating at TB increased, and the integrity of the coating was better maintained after 600 s of friction compared to the original coating without friction, as shown in Figure 2A1, A2. There is visible cracking of the columnar structure at the TP, but no serious detachment has occurred. The EDS elemental analysis of the surface at the friction track after laser texturing treatment for MAO ceramic coating at TB and TP, respectively, under the condition of defatted sheep blood is shown in Figures 5C, D. It was shown by EDS analysis that at TB, the surface elements and the weight ratios were Mg, 23.9 wt%; Si, 13 wt%; O, 40.4 wt%; Al, 1.1 wt%; Ca, 1.1 wt%; and C, 15.2 wt%. The Mg and Al elements are mainly derived from the substrate, and the oxides of Mg and Al are produced during MAO processing. The elements Si and O are mainly derived from the MAO electrolyte, respectively, and the anions move to react with the cations of the substrate in reactions (2) and (3) to produce MgSiO_3_ and Mg_2_SiO_4_, respectively. At the TP region, the surface elements and weight ratios are Mg, 24.4 wt%; Si, 9.8 wt%; O, 40.6 wt%; Al, 1.3 wt%; Ca, 1.0 wt%; C, 15.7 wt%; Na, 0.5 wt%; and F, 3.1 wt%. It is noteworthy that the percentage by mass of elemental Mg at the TP increases while the silicon content decreases. During the MAO reaction stage, it is more reactive at the TP; thus, more SiO_2_ is converted to MgSiO_3_, and the results are consistent with XRD patterns. The parts of the C and O elements are derived from friction and trace residues of defatted sheep blood and rubber gloves. Comprehensively, the MAO coating grows well on the surface after laser texturing and has wear-resistant properties and the ability to resist deformation.

**FIGURE 6:**
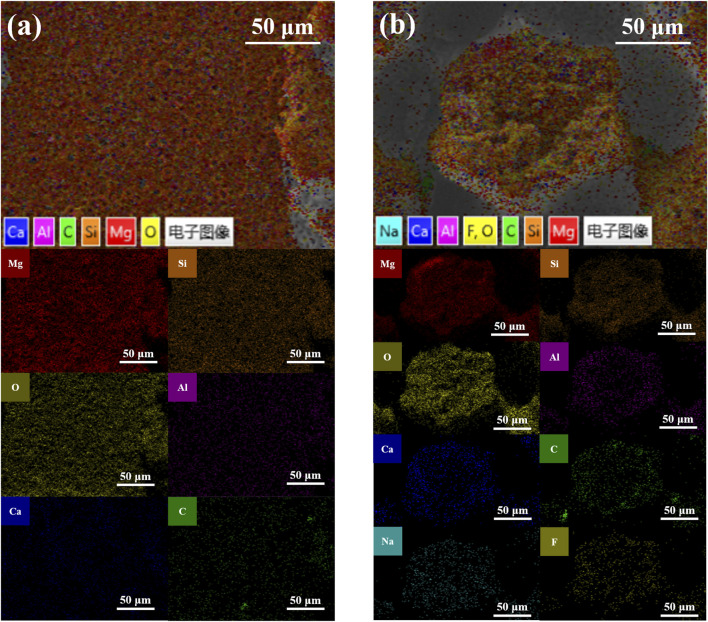
Elemental assignments at the friction trajectory of MAO ceramic coatings performed after the laser texturing process **(A)** at TB and **(B)** at TP.

Figures 6A, B show the results of the distribution of different elements in the TB and TP regions of the MAO ceramic coatings performed after laser texturing processing, respectively. The elements are consistently distributed on the surface of the coating. Interestingly, the electronic images show differences in the presence of the F element. The fact that F is present in blood only in minute quantities has the implication that during the MAO reaction, there is an ability to capture F at the TP to form fluoride (Yu et al., 2019; Wang et al., 2023).

### 3.4 Frictional characteristics during simulated operating conditions

**FIGURE 7:**
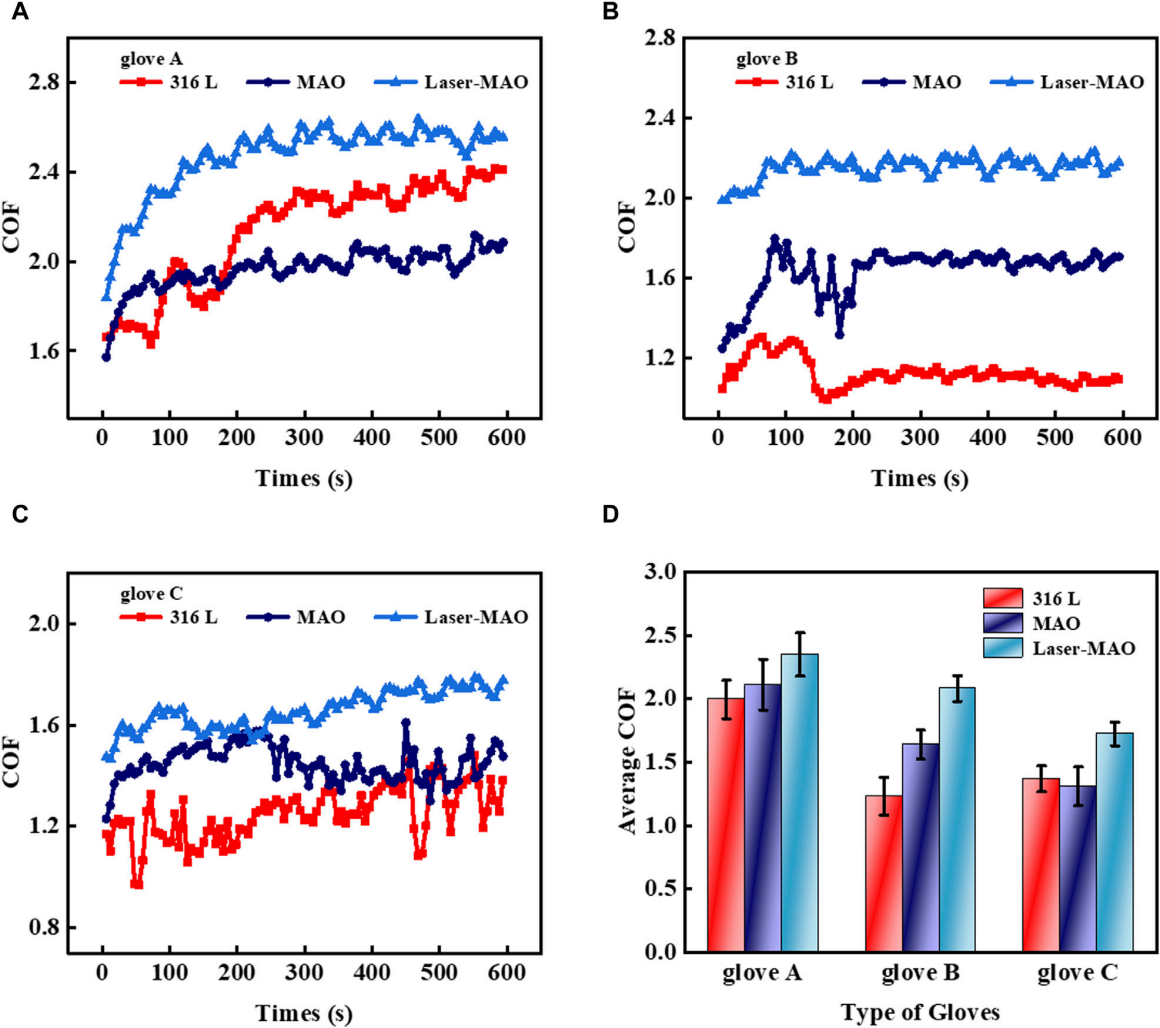
Transient COF of different types of gloves on MAO and Laser-MAO samples under dry friction conditions **(A)** glove A, **(B)** glove B, and **(C)** glove C. **(D)** Average COF between the different types of gloves on MAO and Laser-MAO samples of gloves.

Understanding the frictional interactions between the glove and coating surface is very important as it relates to the suitability of the coating. It is also valuable to assess the variability of these frictional interactions in different working environments, explore the issue of clarifying the frictional properties of different working conditions, and prevent devices from falling and slipping out of hand. Tribology under dry friction conditions simulates the friction that occurs when the sample directly comes into contact with the glove without the addition of lubricants or other fluids. At this point, the action is mainly carried out by the force of action and by the gloves and the tiny, raised portions of the surface of the sample. Figure 7A shows the instantaneous COF of glove A on 316L, untextured, and textured ceramic coatings under dry friction conditions. At the beginning of friction, the instantaneous COF is in the same range. After that, it starts to undulate, which takes longer for the transient COF to maintain a steady state on the smooth 316 L surface. On the MAO ceramic coating, the instantaneous COF tends to level off in the shortest time, followed by Laser-MAO. Figures 7B, C show the instantaneous COF of gloves B and C on 316L, untextured, and textured ceramic coatings, respectively, under dry friction conditions. It is also clearly detectable that the friction on the Laser-MAO ceramic coating shows a tendency to increase. It is worth noting that the instantaneous COF of glove C has fluctuated a lot. This may be due to the better softness and elasticity of glove C, as shown in Table 1. The lowest value of elongation at break for glove C was 840%, while the lowest values for gloves A and B were 800% and 820%, respectively. Figure 7D shows the average COF of the three types of gloves on 316L, untextured, and textured ceramic coatings, which all exhibit an average COF of the Laser-MAO samples greater than that of the MAO ceramic coatings and the smooth 316 L surface. The average COF of the Laser-MAO ceramic coating with the three types of gloves was 2.3, 2.1, and 1.7, respectively. This is mainly due to the presence of large protrusions on the surface of the ceramic coating as a result of laser texturing, which leads to increased transverse shear forces during friction, eventually manifesting themselves in larger values of the COF.

**FIGURE 8:**
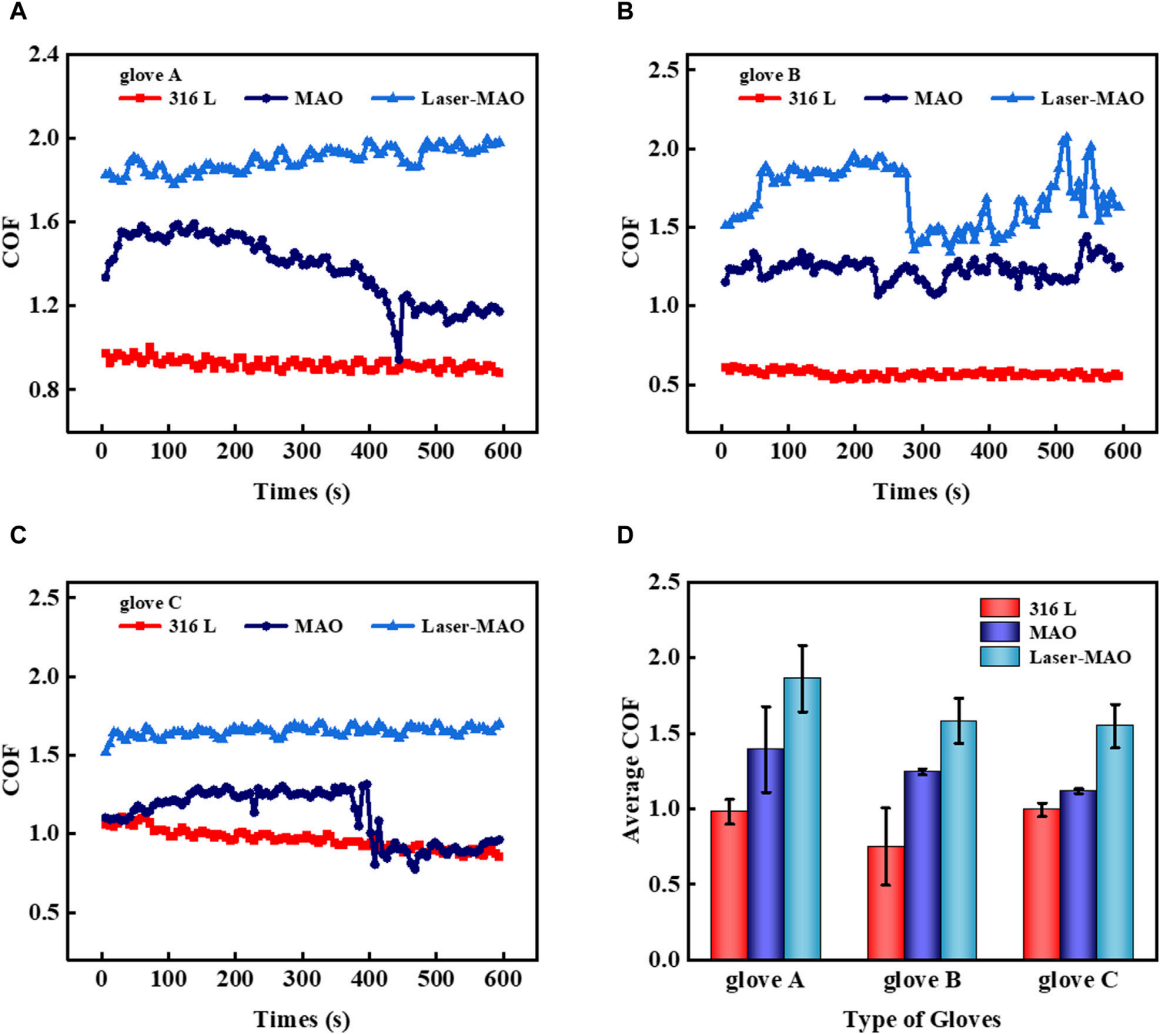
Transient COF of different types of gloves on MAO and Laser-MAO samples under physiological saline conditions **(A)** glove A, **(B)** glove B, and **(C)** glove C; **(D)** average COF between the different types of gloves on MAO and Laser-MAO samples of gloves.

Physiological saline is often used in physiological experiments or clinically owing to its similar osmotic pressure to that of human plasma and tissue fluids. The friction experiments were controlled in an environment of adequate saline (0.9 wt% NaCl aqueous solution) in order to simulate the same plasma and tissue fluid environment as in surgery. Figure 8A shows the instantaneous COF of glove A on 316 L, untextured, and textured ceramic coatings in a physiological saline environment. The instantaneous COF in the saline environment on the surface of the 316 L disks with the glove was kept in a more stable state compared to dry friction. It is due to the fact that in the liquid-lubricated state, the tension of the liquid acts on the glove, making it fit more closely to the mold of the friction machine tool. Figure 8B shows the transient COF of glove B in a saline environment, with a large float in the transient COF of friction on the laser-textured MAO ceramic coating. Figure 8C shows the transient COF of glove C in a saline environment, and the transient coefficient of friction of MAO ceramic coating and glove tends to be the same as that of 316 L. It is notable that glove B does not show the same smooth curve as glove A when rubbed with MAO and Laser-MAO ceramic coatings. This may be a result of the processes used to make the gloves by different manufacturers and the texture of the glove surface. In order to control the variables, the gloves used were commercially available, with a surface characterized as a powder-free flax surface, and only the parameters of the gloves were provided by the merchant and the product test report. However, each of the three gloves showed the same tendency on different friction surfaces. Combining the average COF of the three types of gloves on 316 L, untextured, and textured ceramic coatings in Figure 8D, each of the three types of gloves exhibits the largest average COF between the mating pairs for Laser-MAO, followed by MAO ceramic coatings, and the smallest for 316 L smooth surfaces. In conclusion, laser texturing has the effect of increasing friction.

**FIGURE 9:**
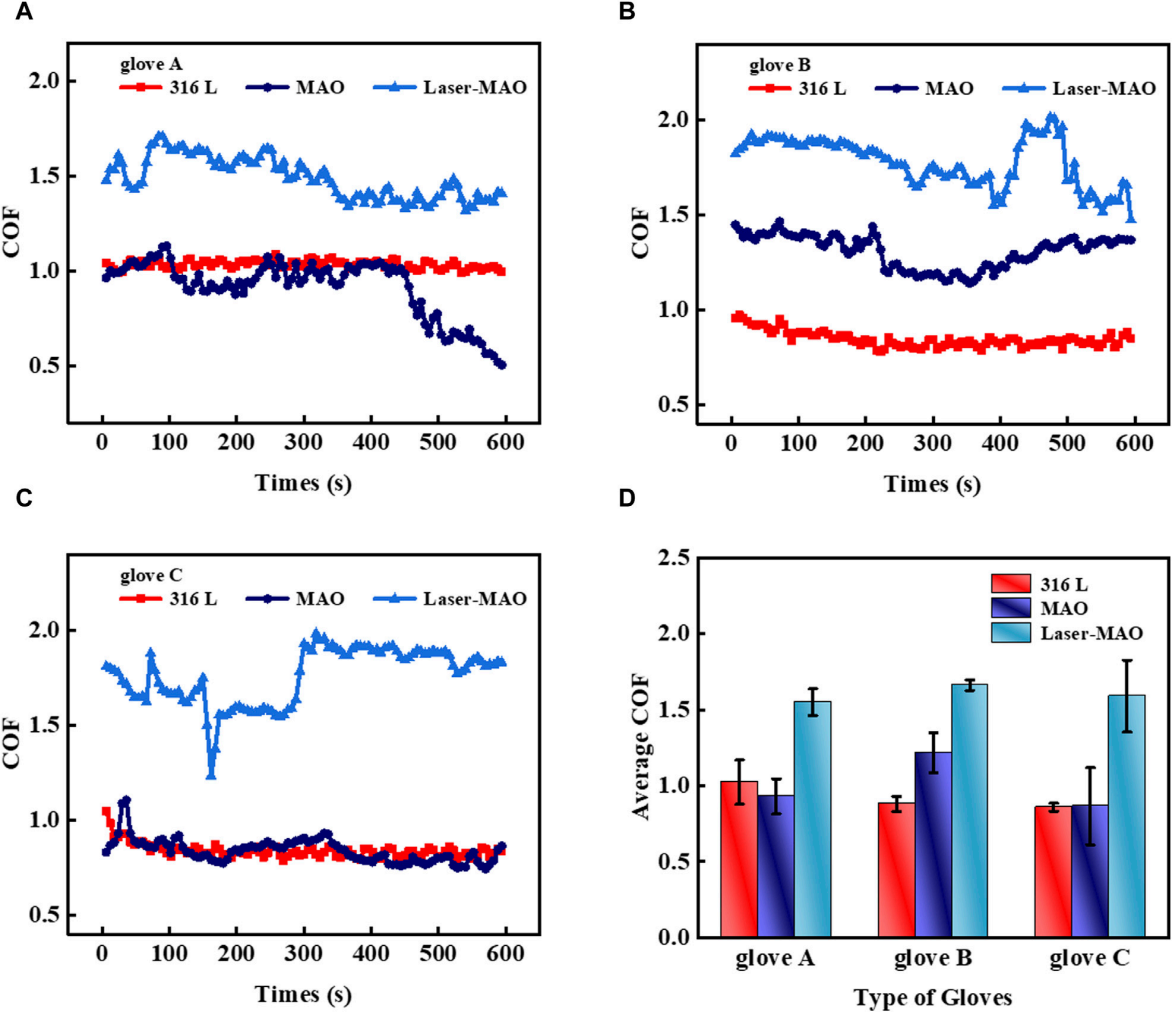
Transient COF of different types of gloves on MAO and Laser-MAO samples under defatted sheep blood conditions **(A)** glove A, **(B)** glove B, and **(C)** glove C; **(D)** average COF between the different types of gloves on MAO and Laser-MAO samples of gloves.

The insertion of physiological saline can only simulate the friction experiment between gloves and samples under the same osmotic pressure, but blood is also less fluid than physiological saline, especially when heat is generated at the point of contact during friction, which makes the blood appear to be coagulated. Easily obtainable fresh defatted sheep blood was inserted into the friction experiments to simulate the COF between the glove and sample in a real blood environment, and the samples were placed in an ice-water bath to maintain ambient temperature as the heat generated under this friction condition is much greater than that generated by a human handgrip. Figures 9A–C show the instantaneous COF of gloves A, B, and C on 316 L, untextured, and textured ceramic coatings in a defatted sheep blood environment. It is evident that the laser texture acts to increase friction. Figure 9D shows the average COF of the three gloves on 316 L, untextured, and textured ceramic coatings. The COF of the gloves on the MAO ceramic coating was between 1.5 and 1.7, which is at the lower value of the average coefficient of friction as compared to dry friction and physiological saline. This is mainly due to the fact that blood has a greater viscosity and is less fluid compared to aqueous solutions, and as a general rule, its frictional resistance should be greater than when it is in a saline solution environment. There are many different types of cells present in the blood, as well as various types of salts and proteins that can act to separate contact surfaces during friction. Meanwhile, during the blood friction process, it is inevitable that heat will be generated in the contact area, making the presence of a few solid particles in the blood, which in the friction makes the sliding friction change into local rolling friction, producing the result of a lower value of the coefficient of friction.

### 3.5 Mechanism analysis

**FIGURE 10:**
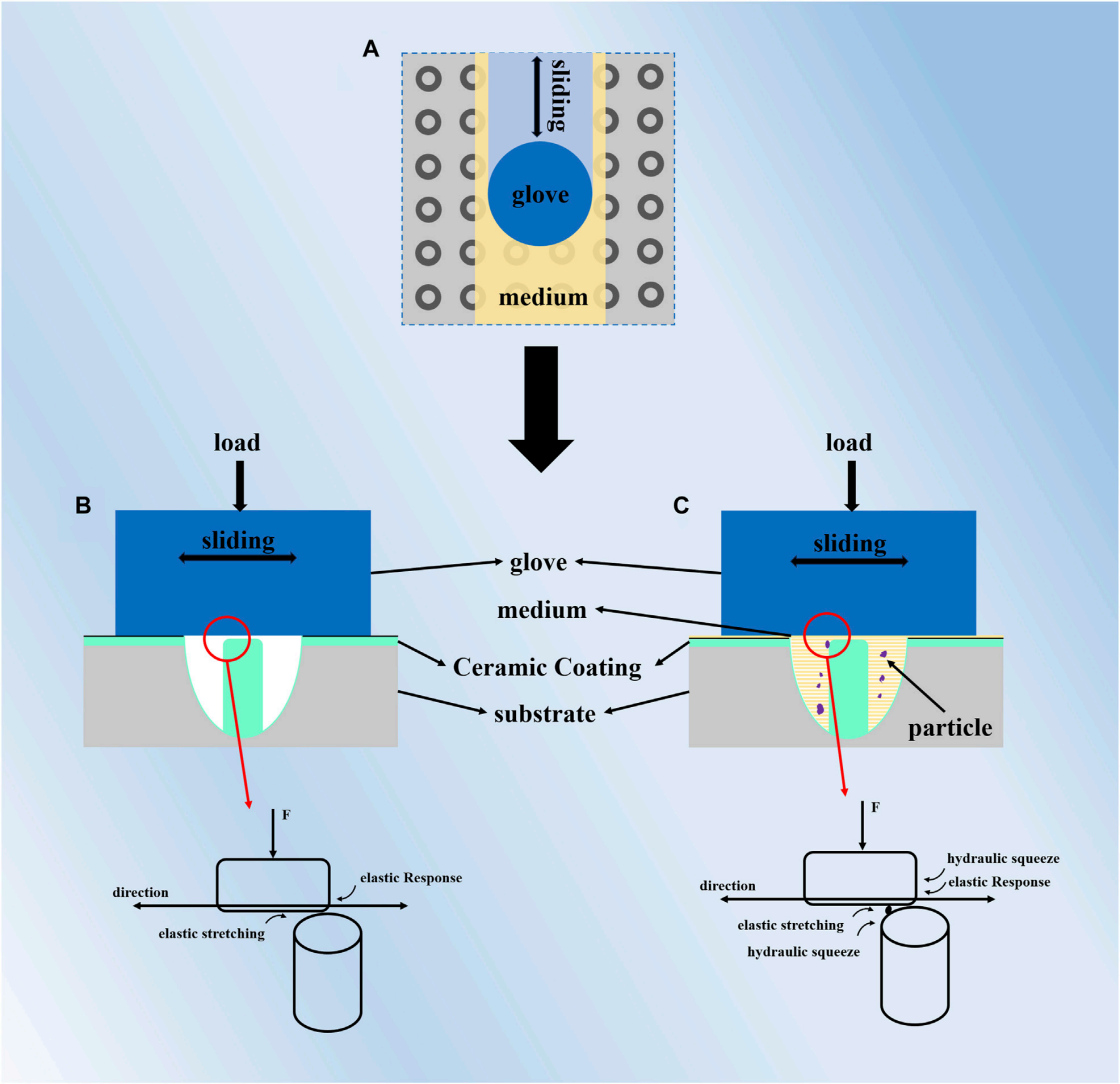
**(A)** Friction schematic; **(B)** friction mechanism under dry friction conditions; **(C)** friction mechanism in the presence of a liquid medium.

Figure 10A shows a schematic diagram of the friction between disposable surgical gloves and Laser-MAO ceramic coating. The slices of gloves were fastened in circular molds. For this experiment, friction experiments were carried out in three environments, according to which the friction media were divided into two cases discussed, one in a dry friction environment and the other in a liquid medium. Figure 10B shows the friction mechanism of disposable surgical gloves with Laser-MAO ceramic coating in a dry friction environment. There is no liquid lubrication between the two friction surfaces under dry friction conditions, and there is increased friction due to the micropores and microcracks on the surface of the MAO ceramic coating, which increases the roughness compared to the 316 L surface. Although the rubber glove slides over the Laser-MAO ceramic coating, there is an elastic stretch between the gloves in the direction of friction traveling, and the existence of opposing forces hinders the glove’s action. The rubber material has a relaxation hysteresis phenomenon that cannot be restored to its original state in time. The movement of the glove is accompanied by the elastic stretching and recovery of the material, which affects the stability of the instantaneous COF. The load applied by the loading rod is vertical, so the elastomer is squeezed and deformed. With the elastic properties of rubber gloves, when the distance between the friction vices is small enough to match the radius of molecular gravitational force, intermolecular forces will produce van der Waals force. In order to overcome the intermolecular interaction, which makes the surfaces of the objects slide against each other, the shear force at the TP of the columnar structure in the rubber and annulus array texture is increased. In the absence of lubricant, there is no lubrication film between the friction parts, which makes the adhesion increase, so the COF increases. Figure 10C shows the friction mechanism of disposable surgical gloves with Laser-MAO ceramic coating under a liquid lubrication medium, which was further divided into physiological saline and defatted sheep blood environments. In a liquid environment, the columnar structure in the middle of the annulus structure changes the direction of movement of the internal liquid during relative movement and acts in the opposite direction on the micro-bump contact points to impede the movement of the friction partner. Defatted sheep blood is more viscous and less mobile, with some carrying capacity to isolate the friction paraphernalia; therefore, its COF is lower than physiological saline. However, there is a certain amount of cellular particles in defatted sheep blood, and there is some blood coagulation during friction, which results in a buildup in the texture, impeding the movement of the fluid and increasing the COF between the friction substituents.

## 4 Conclusion

The annular array texture was produced on the surface of the AZ31 Mg alloy using the laser technique, and MAO ceramic coatings were obtained in a two-stage constant current mode for the electrolyte in the silicate system. The porosity of the MAO ceramic coating is 14.3%, and the porosity of the Laser-MAO ceramic coating decreases to 7.8%, which means that the laser high-temperature pretreatment has a beneficial effect on the growth of micropores with small pores. XRD and EDS results show that the coating grows densely, the elements are uniformly distributed, and there is no obvious change in the coating after friction, which has good wear resistance. Under the action of the annular array texture with the lubrication conditions of physiological saline and defatted sheep blood, all three gloves had a higher average COF on Laser-MAO ceramic coatings than on 316 L and MAO ceramic coatings. Especially in glove C gloves, Laser-MAO ceramic coating on the average COF increased by 86% compared to the other two. The experimental results obtained were in accordance with the expectations and hypotheses. Ceramic coatings combining surface texture and micro-arc oxidation to form an array of annular structures have the effect of increasing frictional resistance. This study is a fusion of the two processes, combining their advantages to form coatings with microstructures and creating friction tests in different friction environments to provide an effective reference for tribological applications. During practical use, it helps prevent the scalpel from falling off and improves the operator’s grip. This shows the combined properties of friction and commercial supplies to form a unique biomaterial with good friction-enhancing and anti-wear capabilities.

## Data Availability

The original contributions presented in the study are included in the article/Supplementary Material; further inquiries can be directed to the corresponding author.
