# Corrigendum: Tribological properties of MAO ceramic coatings with annulus array texture on disposable surgical gloves

**DOI:** 10.3389/fbioe.2024.1470482

**Published:** 2024-09-05

**Authors:** Jing Ji, Zhenbo Bai, Jinfeng Wang, Huiyun Yang, Hailin Lu, Jing Fang

**Affiliations:** ^1^ Department of Gynecology and Obstetrics, The First Affiliated Hospital of Xi’an Jiaotong University, Xi’an, Shaanxi, China; ^2^ Group of Mechanical and Biomedical Engineering, College of Mechanical and Electronic Engineering, Xi’an Polytechnic University, Xi’an, Shaanxi, China

**Keywords:** annulus array texture, micro-arc oxidation, coefficient of friction, disposable surgical gloves, biomaterials

In the published article, the **Reference** for Vencl et al., 2019 was incorrectly written as “Vencl, A., et al. (2019). “SURFACE TEXTURING FOR TRIBOLOGICAL APPLICATIONS,” in A REVIEW SERBIATRIB ‘19 16th International Conference on Tribology in Industry, USA, 17–19 May 2023 (IEEE), 15–17.” It should be “Vencl, A., Ivanović, L., Stojanović, B., Zadorozhnaya, E., Miladinović, S., Svoboda, P. SURFACE TEXTURING FOR TRIBOLOGICAL APPLICATIONS: A REVIEW, Proceedings on Engineering Sciences, 2019, 1(1), pp. 227–239. DOI: 10.24874/PES01.01.029.”

In the published article, the **Reference** for Wang et al., 2023 was incorrectly written as “Wang, J., et al. (2023). Corrosion resistance and biodegradability of micro-arc oxidation coatings with the variable sodium fluoride concentration on ZM21 magnesium alloys. J. Alloys Compd., 962. doi: 10.1016/j.jallcom.2023.171172.” It should be “Wang, J., Dou, J., Wang, Z., Hu, C., Liu, J., Yu, H., Chen, C. (2023). Corrosion resistance and biodegradability of micro-arc oxidation coatings with the variable sodium fluoride concentration on ZM21 magnesium alloys. J. Alloys Compd., 962. doi: 10.1016/j.jallcom.2023.171172.”

In the published article, the **Reference** for Jia et al., 2024 was incorrectly written as “Jia, E., et al. (2024). Study on the negative effects of low sodium hexametaphosphate concentration on MAO coating. Mater. Chem. Phys., 311. doi: 10.1016/j.matchemphys.2023.128552.” It should be “Jia, E., Wang, J., Liu, Q., Xu, G., Lu, H., Peng, Z. (2024). Study on the negative effects of low sodium hexametaphosphate concentration on MAO coating. Mater. Chem. Phys., 311. doi: 10.1016/j.matchemphys.2023.128552.”

The authors apologize for these errors and state that this does not change the scientific conclusions of the article in any way. The original article has been updated.

